# Earthquake Impact on Active Margins: Tracing Surficial Remobilization and Seismic Strengthening in a Slope Sedimentary Sequence

**DOI:** 10.1029/2019GL082350

**Published:** 2019-06-12

**Authors:** Ariana Molenaar, Jasper Moernaut, Gauvain Wiemer, Nathalie Dubois, Michael Strasser

**Affiliations:** ^1^ Institute of Geology University of Innsbruck Innsbruck Austria; ^2^ MARUM‐Center for Marine Environmental Sciences University of Bremen Bremen Germany; ^3^ Surface Waters‐Research and Management Eawag, Swiss Federal Institute of Aquatic Science and Technology Dübendorf Switzerland; ^4^ Department of Earth Sciences ETH Zürich Zürich Switzerland

**Keywords:** surficial remobilization, seismic strengthening, sediment transport, slope stability, Japan Trench

## Abstract

Strong earthquakes at active ocean margins can remobilize vast amounts of surficial slope sediments and dynamically strengthen the margin sequences. Current process understanding is obtained from resulting event deposits and low‐resolution shear strength data, respectively. Here we directly target a site offshore Japan where both processes are expected to initiate, that is, at the uppermost part (15 cm) of a sedimentary slope sequence. Based on a novel application of short‐lived radionuclide data, we identified, dated, and quantified centimeter‐scale gaps related to surficial remobilization. Temporal correlation to the three largest regional earthquakes attest triggering by strong earthquakes (*M*_*w*_ >8). Also, extremely elevated shear strength values suggest a strong influence of seismic strengthening on shallow sediments. We show that despite enhanced slope stability by seismic strengthening, earthquake‐induced sediment transport can occur through surficial remobilization, which has large implications for the assessment of turbidite paleoseismology and carbon cycling at active margins.

## Key Points

- Slope stratigraphy reveals direct impact of strong earthquakes on young sediments of the active margin offshore Japan
- Remobilization of surficial slope sediments at site occurs during *M*_*w*_ 8+ earthquakes, allowing turbidite paleoseismology in terminal basins
- Seismic strengthening is most effective on uppermost sediments, progressively reducing submarine landslide potential

## 1 Introduction

Understanding and quantifying earthquake impact on active margin slope sequences are crucial for our knowledge on sedimentary processes and geomorphological evolution of subduction zones. These processes form the link between seismic shaking and the depositional record in slope basins and subduction trenches, which has been analyzed for a wide range of scientific disciplines, such as volcanic eruptions (e.g., Ikehara et al., 2017), submarine landslides (e.g., Ikari et al., 2011), turbidite paleoseismology (e.g., Goldfinger et al., 2012), and carbon supply to the deep sea (e.g., Thunell et al., 1999). Previous work discovered the potential importance of two seismically controlled processes involving sediment transport and affecting slope stability, termed surficial remobilization (McHugh et al., 2016; Moernaut et al., 2017), and seismic strengthening, respectively (Lee et al., 2004).

Surficial remobilization involves the removal of the uppermost centimeters of slope sediment by seismic shaking. Recent studies proposed this process by documenting high‐turbidity bottom waters following strong earthquakes (e.g., Ashi et al., 2014; Noguchi et al., 2012), by identifying very young sediments within fine‐grained seismo‐turbidites (e.g., McHugh et al., 2016; Moernaut et al., 2017) and by comparing the volume of earthquake‐induced deposits with slope sediment recharge rates (Goldfinger et al., 2017). Submarine landsliding involves translational or rotational sliding of “thick” sediment packages (meter‐scale) and is highly dependent on the disposition of geotechnically “weak layers,” buildup of critical overburden stress, and excess pore pressure (Locat & Lee, 2002). In contrast, surficial remobilization is thought to be controlled by earthquake‐induced transient stresses at the sediment‐water interface, remolding the upper veneer of surficial sediment (Moernaut et al., 2017). This fundamental difference in remobilization depth is potentially crucial for the feasibility of turbidite paleoseismology as surficial remobilization does not require “weak layers”, excess pore pressure, and sufficient time to recharge slope sedimentary sequences. This could explain the continuity of turbidite paleoseismic records in several settings despite a scarcity of observed landslides (e.g., Goldfinger et al., 2012; Patton et al., 2013; Pouderoux et al., 2014) or lack of active sediment recharge on slopes (Goldfinger et al., 2017). Furthermore, seismically driven surficial remobilization has been suggested as an important process for carbon supply to the hadal zone (Kioka et al., 2019) due to the enhanced concentration of organic matter in surface sediments (Burdige, 2007). However, most studies on earthquake‐induced sediment transfer based their process assessment solely on the characterization of the final products, that is, the turbidite records in depositional basins (e.g., Ikehara et al., 2016; McHugh et al., 2016). Because such depositional records can be influenced by a wide range of processes during sediment transport and deposition, it remains equivocal to fully characterize the underlying remobilization process solely based on these deposits.

Seismic strengthening relates to enhanced sediment compaction (i.e., reduced void ratio) through seismic shaking (Lee et al., 2004). Several studies propose that the consolidation state of a sedimentary sequence can be estimated using the downcore trend of undrained shear strength (*S*_*u*_) normalized to the effective overburden stress (σ′_v0_). Sedimentary sequences along active margins typically show much higher normalized *S*_*u*_ values than passive margins, which has been explained by seismic strengthening (e.g., Sawyer & DeVore, 2015). Seismically enhanced *S*_*u*_ would result in higher slope stability, which may explain the scarcity of landslide occurrence observed along subduction zones (e.g., Strozyk et al., 2010; ten Brink et al., 2016). So far, *S*_*u*_ compilations only include long sequences (100 m) in a rather low data resolution (~1 point per m; Sawyer & DeVore, 2015). However, seismic strengthening is expected to be most effective on the uppermost loosely packed sediments as repeated cyclic loading experiments show the strongest void ratio reduction associated to the first shaking events (Lee et al., 2004).

It thus seems that our understanding of both earthquake‐triggered surficial remobilization and seismic strengthening suffers from the absence of studies targeting the location where these processes take place, that is, the uppermost part of the sedimentary slope sequence. Therefore, we focus on recent sediments on an active margin slope and select the Japan Trench margin as an ideal case study where both earthquake‐triggered surficial remobilization and seismic strengthening are proposed (McHugh et al., 2016; Sawyer & DeVore, 2015). We investigate surficial remobilization by identifying, dating, and quantifying centimeter‐scale gaps in the slope stratigraphy using a novel approach based on xs^210^Pb activity profiles. We evaluate the potential for earthquake triggering by temporal correlation with the regional earthquake record. Also, we assess seismic strengthening by analyzing the normalized *S*_*u*_ of the surficial slope sediment. Hereby, our study will investigate—for the first time—the different impacts of earthquake shaking on young sediments on an active margin slope.

## 2 Study Area

**Figure 1:**
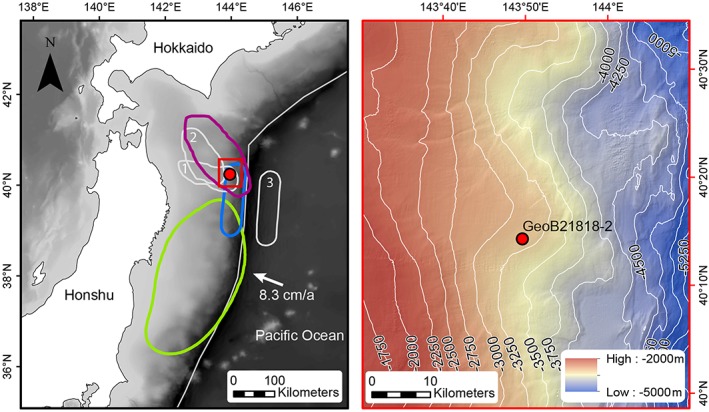
(left) Overview map of NE Japan Trench subduction margin with rupture areas of major historical earthquakes and location of coring site GeoB21818‐2 (red dot). green: 2011 CE Tohoku‐oki (M_w_ 9.1; (Wang & Bilek, 2014), purple: 1968 CE Tokachi‐oki (M_w_ 8.2; Lay, 2018), and blue: 1896 CE Sanriku‐oki “tsunami earthquake” (M_w_ 8; Satake, 2017). The grey lines indicate the rupture areas of three other large earthquakes: 1.: 1994 CE (M_w_ 7.7), 2.: 1931 CE (M_w_ 7.8) earthquake (Lay, 2018), and 3.: 1933 CE (M_w_ 8.4) outer‐rise earthquake (Satake, 2017). (right) Bathymetric map with coring site GeoB21818 on a broad ridge on the midslope of the Japan Trench margin. The bathymetric map was compiled using data from Japanese cruises and R/V Sonne SO251‐1 cruise (Hydrographic and Oceanographic Department, Japan Coast Guard & JAMSTEC, 2011; Strasser et al., 2017).

Core site GeoB21818 (40.2465°N, 143.8135°E) is located at the NE Japan Trench margin where the Pacific Plate subducts beneath the Okhotsk Plate with an average convergence rate of 8.3 cm/a (Figure 1). Several large historical earthquakes ruptured this area, of which the 1968 CE Tokachi‐oki (*M*_*w*_ 8.2) earthquake and the 1896 CE Sanriku‐oki (*M*_*w*_ 8) tsunami earthquake are the largest. Three other significant earthquakes in this region were the 1933 CE Sanriku‐oki (M_w_ 8.4) outer‐rise earthquake (Kanamori, 1971) as well as the 1994 CE Sanriku‐oki (M_w_ 7.7) and 1931 CE (*M*_*w*_ 7.8) interplate earthquakes (Yamanaka & Kikuchi, 2004). The 2011 CE Tohoku‐oki (*M*_*w*_ 9.1) earthquake occurred south of our study site. To compare observations for different earthquakes, we aimed for a slope record potentially containing multiple imprints of surficial remobilization. Therefore, we chose a coring site north of the 2011 CE Tohoku‐oki rupture area as this earthquake severely impacted its rupture area in terms of sediment remobilization (e.g., Arai et al., 2013; McHugh et al., 2016). Toward our study area (~45 km south of site GeoB21818), only small deposits related to the 2011 CE Tohoku‐oki earthquake were identified (McHugh et al., 2016). Our coring site GeoB21818 is located at 3,138‐m depth and about 165‐km offshore to avoid the effects of coastal processes and wave action. Moreover, it is situated on a 2.5° dipping shoulder to avoid erosion and/or deposition related to sediment gravity flows originating upslope.

## 3 Methods

### 3.1 Sediment Core Analyses

Core GeoB21818‐2 is a pilot gravity core (15‐cm length) taken during research cruise SO251‐1 onboard RV Sonne (Strasser et al., 2017) using a piston coring system provided by JAMSTEC. No free‐fall was used to obtain an undisturbed sample of the sediment‐water interface. Continuous sampling at 1‐cm resolution was performed for radionuclide measurements. Measurement of xs^210^Pb and ^137^Cs activity was conducted at the EAWAG (Dübendorf, Switzerland) using *CANBERRA* and *Princeton Gamma‐Tech* germanium well detectors with 2‐ to 3‐day measurement time per sample. The xs^210^Pb‐derived age model was calculated using the constant initial concentration model assuming constant xs^210^Pb concentration upon sedimentation (Cundy & Croudace, 1995; Sanchez‐Cabeza & Ruiz‐Fernández, 2012). More information on the choice of xs^210^Pb age model can be found in the supporting information. Grainsize data were obtained using laser diffraction with a *Malvern Mastersizer 2000* after 1‐min ultrasonification. Grainsize distribution statistics were computed with the GRADISTAT software (Blott & Pye, 2001) using the Folk and Ward (1957) graphical method. *S*_*u*_ was calculated to estimate the sediments consolidation state by fall cone penetrometer measurements conducted directly after core opening at MARUM (University of Bremen) using a cone with a defined weight of 79.8 g, opening angle of 30° and cone factor of 0.85 (Wood, 1985). For normally consolidated diatom‐rich sediment, we used a *S*_*u*_/σ′_v0_ range of 0.2‐0.5 (as described by Wiemer et al., 2017). σ′_v0_ was calculated assuming hydrostatic conditions using *σ*′_*v*0_ = *γ* ′  * *z*, with *γ*′ as submerged unit weight of 3.9 kN/m^3^ based on bulk density of 1.4 g/cm^3^ (Noguchi et al., 2012) and *z* as subbottom depth. Sediment with *S*_*u*_ >0.5 * σ′_v0_ is considered as overconsolidated and with *S*_*u*_ <0.2 * σ′_v0_ as underconsolidated. X‐ray computed tomography (CT) scans were taken 4 months after core opening using a *Siemens SOMATOM Definition AS* (Medical University Innsbruck) with 0.23‐ × 0.23‐ × 0.30‐mm resolution. CT data visualization and analyses were conducted using the program FIJI (Schindelin et al., 2012). A radiodensity profile was obtained by averaging over the cross section of the working half section.

### 3.2 Strategy for Identification of Surficial Remobilization and Seismic Strengthening

**Figure 2:**
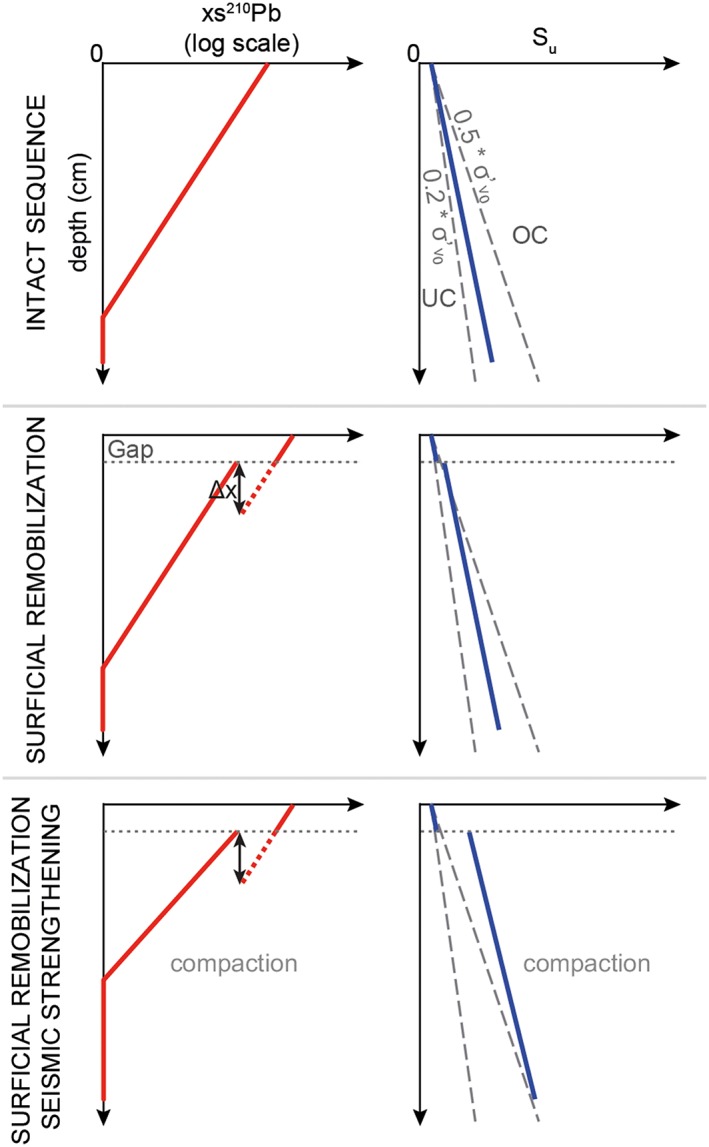
Hypothetical profiles of xs^210^Pb activity (logarithmic scale) and S_u_ of a sedimentary slope sequence in three different scenarios. The dashed grey lines in the S_u_ profiles outline the normal consolidation domain represented by the range 0.2‐0.5 * σ′_v0_ starting at first S_u_ value to account for cohesion. UC: underconsolidation, OC: (apparent) overconsolidation. Δx represents the remobilization depth, which caused the gap in the xs^210^Pb activity profile.

Visual detection of gaps is not possible at site GeoB21818 as sediment is bioturbated and shows no lamination (Strasser et al., 2017). Here we propose a new strategy using xs^210^Pb activity to identify, quantify, and date centimeter‐scale gaps in slope sequences. Figure 2 shows hypothetical profiles of xs^210^Pb activity and *S*_*u*_ for the upper few centimeters of a slope sequence in three different conditions: (i) intact and undisturbed, (ii) affected by surficial remobilization, and (iii) affected by both surficial remobilization and seismic strengthening.

In an intact and undisturbed sequence without redistribution of xs^210^Pb, variability in accumulation rate, or xs^210^Pb flux variation (Sanchez‐Cabeza & Ruiz‐Fernández, 2012), xs^210^Pb activity decays monotonically and exponentially with depth displayed as a straight line on a logarithmic scale. Sedimentation rate (SR) can be determined using the xs^210^Pb activity versus depth profile by SR =  − *λ*/*b*, with *λ* being the ^210^Pb radioactive decay constant and *b* a constant in the function fitted to the xs^210^Pb activity profile (i.e., *xs*^210^*Pb* = *c* * *e*^*b* * *depth*^) (Arnaud et al., 2002). Consolidation, compaction and erosional processes can affect the obtained SR. Therefore, SR does not fully represent the actual amount of sediment deposited at the ocean floor (accumulation rate). *S*_*u*_ in an intact sequence increases linearly by increasing sediment load and lies within the normal consolidation range (0.2‐0.5 for *S*_*u*_/σ′_v0_).

According to our hypothesis, a slope sequence affected by one surficial remobilization event will show a jump in the xs^210^Pb decay curve, termed gap, as a few centimeters of surficial sediment are removed. Normal sedimentation is assumed to continue directly after the remobilization event. As bioturbation and large variation in accumulation rate or xs^210^Pb flux can cause local xs^210^Pb fluctuations (Appleby, 1998), we only consider gaps bordered by sections with a distinct xs^210^Pb activity decrease over multiple samples. We expect *S*_*u*_ of the slope sequence to be slightly higher, but within the normal consolidation range. With increasing overburden stress applied by postevent sedimentation, the jump in *S*_*u*_ gets smaller and disappears once the preevent overburden stress is exceeded.

A slope sequence affected by surficial remobilization and seismic strengthening is also expected to have a jump in the xs^210^Pb profile. However, due to seismically induced compaction of the lower section, the xs^210^Pb activity below the gap would decrease faster with depth, exhibiting a lower SR. *S*_*u*_ values would show a significant increase and plot into the overconsolidation domain. This *S*_*u*_ jump would disappear once the overburden stress increases sufficiently for the *S*_*u*_ to fall again in the normal consolidation range (0.2‐0.5 * σ′_v0_).

Accordingly, if data follow the hypothetical profiles and overall sedimentation is continuous, the gap can be dated by extrapolation of the age‐depth model of the upper section.

Furthermore, this framework allows for quantification of the remobilization depth (i.e., the amount of sediment removed to cause the observed gap; Δx in Figure 2). First, the xs^210^Pb activity at the gap can be extrapolated from the lower section of the xs^210^Pb activity profile. Next, to obtain the depth of the extrapolated xs^210^Pb activity in an intact sequence, this xs^210^Pb activity is inserted into the xs^210^Pb activity versus depth function of the upper section. The difference between current gap depth and depth of the extrapolated xs^210^Pb activity in an intact sequence represents the remobilization depth assuming that the upper section was not compacted.

## 4 Results

### 4.1 Sediment Core Data

**Figure 3:**
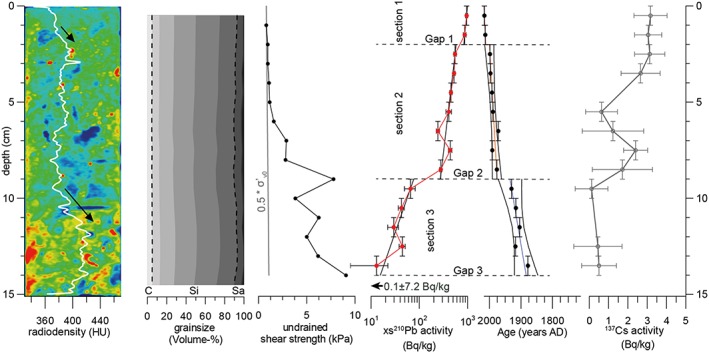
Computed tomography image (blue: low radiodensity; red: high radiodensity) and radiodensity profile (arrows indicate general increase in radiodensity), cumulative grainsize distribution (C: clay, Si: silt, and Sa: Sand; the dashed lines mark borders between the three fractions), S_u_ along with the upper normal consolidation border (0.5 * σ′_v0_) starting at first measurement to account for cohesion or coring‐induced compaction, xs^210^Pb activity (logarithmic scale) and age‐depth model as well as ^137^Cs activity of core GeoB21818‐2. xs^210^Pb activity at 14.5 cm is below the detection limit.

Core GeoB21818‐2 consists of bioturbated diatomaceous mud with a mean grain size of medium to coarse silt and poor sorting (Figure 3). The grainsize distribution shows limited variation throughout the core. CT scan data show abundant bioturbation features represented by lower radiodensity and burrow shapes (i.e., dark blue areas in Figure 3). The uppermost ~1.5 cm is devoid of traceable bioturbation features. Overall, the radiodensity profile shows two units from about 2‐9.5 cm and 11.5‐14 cm with rather constant values of ~380 and ~420 HU, respectively. Increasing radiodensity trends can be observed from ~1‐2 cm and ~9.5‐11.5 cm. For the upper 6 cm, *S*_*u*_ is around 1 kPa and lies close to the normal consolidation domain (0.2‐0.5 * σ′_v0_). At 9 cm, *S*_*u*_ increases sharply into the overconsolidation domain (>0.5 * σ′_v0_) with an average of ~6 kPa down‐core.

Three decreases in xs^210^Pb activity can be observed at (i) 1.5‐2.5 cm, (ii) 8.5‐9.5 cm, and (iii) 13.5‐14.5 cm, which subdivide the xs^210^Pb profile into three sections with a distinct decreasing xs^210^Pb activity trend. In section 3, xs^210^Pb activity decays about a factor of 3 faster with depth than in sections 1 and 2. The xs^210^Pb activity is below the detection limit at 14.5 cm as the level of supported ^210^Pb is reached. xs^210^Pb activity fluctuates (i.e., single point excursions) at 6.5‐7.5 and 12.5‐13.5 cm. ^137^Cs activity forms a plateau from top of core to 3.5 cm and peaks at 7.5 cm. From 9.5 cm downward, ^137^Cs activity lies near the detection limit suggesting deposition before nuclear testing in 1952 CE and agreeing with the xs^210^Pb‐derived age of 1930±32 CE at 9.5 cm.

### 4.2 Identification, Dating, and Quantification of Potential Surficial Remobilization Gaps

In contrast to local xs^210^Pb activity fluctuation (at 6.5‐7.5 cm and 12.5‐13.5 cm), xs^210^Pb activity steps at (i) 1.5‐2.5 cm (G1), (ii) 8.5‐9.5 cm (G2), and (iii) 13.5‐14.5 cm (G3) are bordered by sections of overall monotonic xs^210^Pb decrease. Therefore, we suggest G1, G2, and G3 are actual gaps in the slope sequence for which we calculated the age and remobilization depth (Table 1) following the strategy outlined in section 3.2.

**Table 1:** Sections and Gaps Identified in the xs^210^Pb Profile Along With Major Historical Earthquakes Which Potentially Induced the Gaps

Name	Depth (cm)	Sedimentation rate (mm/a)	Remobilization depth (cm)	Age (CE)	Earthquake	M_w_
Section 1	0‐2	3.5				
Gap 1	2		4	~2012±2	2011 CE Tohoku‐oki	9.1
Section 2	2‐9	2.9				
Gap 2	9		12	1976 ±14	1968 CE Tokachi‐oki	8.2
Section 3	9‐14	1.0				
Gap 3	14		min. 2	1884±37	1896 CE Sanriku‐oki	8

SRs of 3.5, 2.9, and 1.0 mm/a were calculated for sections 1, 2, and 3, respectively. In turn, G1 (at 2 cm), G2 (at 9 cm), and G3 (at 14 cm) were dated to ~2012±2 CE, 1976±14 CE, and 1884±37 CE. The age of G1 must be considered an approximation as section 1 consists of only two measurement points inhibiting calculation of confidence intervals. The error of 2 years is based on the vertical sample size of 1 cm combined with the SR of section 1. Remobilization depths of 4 and 12 cm were calculated for G1 and G2, respectively. For G3, as xs^210^Pb activity at 14.5 cm is below the detection limit and extrapolation of xs^210^Pb activity at the gap is therefore not possible, a minimum remobilization depth of 2 cm was obtained using the maximum xs^210^Pb activity at 14.5 cm (0.1±7.2 Bq/kg).

## 5 Discussion

### 5.1 Earthquake‐Triggered Remobilization of Surficial Slope Sediments

The two largest earthquakes rupturing the area of site GeoB21818 were the 1968 CE Tokachi‐oki (*M*_*w*_ 8.2) and 1896 CE Sanriku‐oki (*M*_*w*_ 8) tsunami earthquake (Figure 1). Both 1968 CE and 1896 CE earthquake fall within the age range calculated for G2 and G3 (1976±14 CE and 1884±37 CE; Table 1), respectively. G1 was dated to ~2012±2 CE suggesting that the 2011 CE Tohoku‐oki (*M*_*w*_ 9.1) earthquake induced this gap. As all three gaps correlate with the largest regional earthquakes, we suggest that earthquake‐triggered surficial remobilization caused the removal of sediment resulting in the gaps. We could not identify a gap associated to the 1994 CE Sanriku‐oki (*M*_*w*_ 7.7), 1931 CE (*M*_*w*_ 7.8) earthquake, and 1933 CE (*M*_*w*_ 8.4) outer‐rise earthquake suggesting that no or only minor remobilization took place for these earthquakes. The 1933 CE earthquake originated in the oceanic plate east of the subduction trench possibly explaining the absence of a remobilization gap as seismic waves may be attenuated when traveling through the shallow plate interface (Usami et al., 2018).

To evaluate whether seismic ground motion parameters control surficial remobilization occurrence, we calculated peak ground acceleration (PGA) at site GeoB21818 for the largest regional earthquakes with the empirical ground motion attenuation relation of Si and Midorikawa (1999), which uses shortest fault distance (Kita et al., 2010), focal depth (Nagai et al., 2001; Satake et al., 2017; Yoshida et al., 2011), earthquake magnitude, and a constant for interplate earthquakes (see the supporting information). In contrast to the 1968 CE (*M*_*w*_ 8.2; PGA ~0.6 g) and 1896 CE (*M*_*w*_ 8; PGA ~0.6 g) earthquakes, the 1994 CE (*M*_*w*_ 7.7; PGA ~0.6 g) and 1931 CE (*M*_*w*_ 7.8; PGA ~0.5 g) earthquakes did not lead to observable remobilization despite all having similar estimated PGAs. However, the more remote 2011 CE (*M*_*w*_ 9.1; PGA ~0.3 g) earthquake with lower PGA did cause subtle remobilization (4 cm). We suggest that mainly earthquake magnitude—and not PGA—controls the occurrence of surficial remobilization and propose a magnitude threshold of ~*M*_*w*_ 8 at site GeoB21818. Earthquake magnitude relates directly to the duration of significant ground motion for such large events (Hanks & McGuire, 1981), and thus the potential for remobilizing larger amounts of surficial slope sediments with increasing magnitude. However, other seismological factors, such as the frequency content of the seismic ground motion at site, may also influence sediment remobilization processes during seismic shaking (Van Daele et al., 2019).

Our data show that xs^210^Pb profiles can be used to identify, date, and quantify small centimeter‐scale gaps in young slope sedimentary sequences, provided that the site of choice is sheltered from sediment gravity flows. Also, it must be noted that earthquakes might “overprint” the gaps of previous events if more sediment is eroded during the most recent event than deposited after the older one. This would lead to an underrepresentation of earthquake events based on gaps within the sequence, and therefore, the paleoseismic potential of remobilization gaps in slope sequences seems rather limited.

### 5.2 Seismic Strengthening at Active Margins

The increase in radiodensity and *S*_*u*_ around 9 cm cannot be explained by changes in lithology or grain size as both remain virtually constant throughout the core. Bioturbation can potentially affect *S*_*u*_ (Locat et al., 2002); however, its ubiquitous presence in both the normally consolidated section 2 as overconsolidated section 3 suggests that bioturbation had no or only minor influence. Therefore, we suggest that a reduction of void ratio caused the elevated *S*_*u*_ and radiodensity corroborated by a faster decay of xs^210^Pb activity in section 3 suggesting compaction. Unroofing by surficial remobilization (G1 and G2) cannot account for the overconsolidated state of section 3 as it only removed a minor amount of overburden stress. Therefore, the sediments' consolidation state should be termed “apparent overconsolidation” as it was not caused by actual overburden of sediment. Instead, we propose seismic strengthening as the cause of apparent overconsolidation in section 3.

The average *S*_*u*_ at 9‐15 cm (~6 kPa) would only be expected at ~3‐ to 7‐m depth in a normally consolidated sequence. Also, the average normalized *S*_*u*_/σ′_v0_ of ~12 is an order of magnitude higher than the range 0.4‐1.0 derived from a compilation of low‐resolution measurements on 100‐m‐long cores in active margins (Sawyer & DeVore, 2015). Therefore, we propose that seismic strengthening is most effective on the uppermost sediment and its impact decreases with burial depth and each successive earthquake.

### 5.3 Implications for Earthquake‐Triggered Sediment Transport and Margin Development

Our study provides first indication of earthquake‐triggered surficial remobilization observed directly on a slope stratigraphy. We identified and quantified three gaps in a slope sequence using the xs^210^Pb activity profile, which ages (within uncertainty) correspond to the three largest earthquakes of the region: the 2011 CE Tohoku‐oki (M_w_ 9.1), 1968 CE Tokachi‐oki (M_w_ 8.2), and 1896 CE Sanriku‐oki (M_w_ 8) earthquakes. Our findings allow pinpointing a magnitude threshold of ~*M*_*w*_ 8 and typical remobilization depths of 4‐12 cm for our specific slope site. This complements previous studies based on the composition of turbidites, which suggested earthquake‐triggered remobilization of the upper 1‐9 cm of slope sediments (McHugh et al., 2016; Moernaut et al., 2017). Furthermore, our results are in line with recent studies discussing the importance of earthquake shaking for transporting organic‐rich surficial slope sediment to the hadal trench, forming an important contributor to the marine carbon cycle (Bao et al., 2018; Kioka et al., 2019; Mountjoy et al., 2018).

Also, our study provides the first high‐resolution (1 cm) indication of seismic strengthening of shallow slope sediments suggesting that both surficial remobilization and seismic strengthening can affect slope sequences during seismic shaking. However, they might affect different stratigraphic levels as surficial remobilization occurs directly at the sediment‐water interface, whereas seismic strengthening possibly affects slightly deeper sediments that are consolidated enough to allow compaction upon seismic shaking. Despite seismic strengthening causing higher slope stability—impeding earthquake‐triggered submarine landslides—our study corroborates that earthquake‐triggered sediment transport at active margins can be driven by the process of surficial remobilization.

## Supporting information



Supporting Information S1

Data Set S1
