# Supplementary material for: Earthquake Impact on Active Margins: Tracing Surficial Remobilization and Seismic Strengthening in a Slope Sedimentary Sequence
Source: Geophys Res Lett. 2019 Jun 12;46(11):6015–23. doi: 10.1029/2019GL082350 (PMC6686709; doi:10.1029/2019GL082350)
Supplement: Supplementary file 1 — Supporting Information S1 [file GRL-46-6015-s001.pdf]

AGU PUBLICATIONS

*Geophysical Research Letters*

Supporting Information for

**Earthquake Impact on Active Margins: Tracing Surficial Remobilization and Seismic Strengthening in a Slope Sedimentary Sequence**

Ariana Molenaar[1], Jasper Moernaut[1], Gauvain Wiemer[2], Nathalie Dubois[3,4], Michael Strasser[1,2]

[1] Institute of Geology, University of Innsbruck, Innsbruck, Austria

[2] MARUM-Center for Marine Environmental Sciences, University of Bremen, Bremen, Germany

[3] Surface Waters – Research and Management, Eawag, Swiss Federal Institute of Aquatic Science and Technology, Dübendorf, Switzerland

[4] Department of Earth Sciences, ETH Zürich, Zürich, Switzerland

**Contents of this file**

Text S1
Text S2
Figure S1
Table S1

**Additional Supporting Information (File uploaded seperately)**

Caption for Dataset 1

**Introduction**

This supporting information provides further details and discussion on different xs$^{210}$Pb age models (Text S1) and on peak ground acceleration calculations at core site GeoB21818 for the five largest regional earthquakes (Text S2). Results and parameters used in the calculations are described and listed in Figure S1 and Table S1.

**Text S1.**

**Further explanation on the $xs^{210}Pb$ age model.**

There are three $xs^{210}Pb$ age modelling techniques commonly used for dating of recent sediments: the constant flux constantsedimentation (CFCS), constant initial concentration (CIC) and constant rate of supply (CRS).

The CFCS model assumes both a constant flux of $xs^{210}Pb$ and sedimentation rate. A sedimentation rate is derived directly from the exponential fit to the $xs^{210}Pb$ activity profile which is used to calculate the ages (Arnaud et al., 2002). Therefore, gaps in the sequence will lead to underestimation of ages if the remobilization depth is not accurately determined.

The CIC model assumes a constant activity of $xs^{210}Pb$ in surface sediment ($C_0$) upon deposition throughout time allowing for direct calculation of ages for each $xs^{210}Pb$ measurement. The formula

$$t(z) = \frac{1}{\lambda} \ln(\frac{C_0}{C_z})$$

is used with *t(z)* as the age at depth *z*, $C_0$ as the initial activity upon deposition and $C_z$ the $xs^{210}Pb$ activity at depth *z* (Sanchez-Cabeza & Ruiz-Fernández, 2012).

The CRS model assumes that the flux of $xs^{210}Pb$ to the sediment is constant through time and sedimentation rate may vary, therefore allowing for variation in $xs^{210}Pb$ activity upon deposition. This model uses the formula

$$t(z) = \frac{1}{\lambda} \ln(\frac{A_0}{A_z})$$

with *t(z)* as the age at depth *z*, $A_0$ as the complete $xs^{210}Pb$ activity inventory and $A_z$ the $xs^{210}Pb$ inventory below depth *z* (Sanchez-Cabeza & Ruiz-Fernández, 2012). As an accurate estimation of the total $xs^{210}Pb$ inventory ($A_0$) is key for this model and we investigate a slope stratigraphy for erosive events, this method is not adequate for our study.

We decided for the CIC model as this age model allows for dating of individual ages independent of stratigraphic gaps. Main assumption of this model is a constant concentration of $xs^{210}Pb$ upon deposition. As $xs^{210}Pb$ is mostly scavenged from the water column by clay to very-fine silt particles (Cundy & Croudace, 1995) which stays more or less constant throughout the core (16-18%), we argue that this assumption is justified for our study site.

**Text S2.**

**Estimation of peak ground acceleration at site GeoB21818.**

Peak ground accelerations (PGA) were calculated following (Usami, Ikehara, Kanamatsu, & McHugh, 2018) using the empirical ground motion attenuation relations of Si and Midorikawa (1999):

$$\log(PGA) = b - \log(x + c) - 0.003x$$

With

$$b = 0.5M_w + 0.0043D + d + 0.61$$
$$c = 0.0055 * \ 10^{0.5M_w}.$$

The formula uses *x* as the shortest distance from the fault plane (i.e. fault distance), $M_w$ as the earthquakes' moment magnitude, *D* as focal depth, and *d* as a constant relating to the type of earthquake (crustal: 0.00, interplate: 0.01 and intraplate: 0.22; Table S1 and Figure S1). We derived the fault distance using Pythagoras' theorem by implementing the shortest horizontal distance from the rupture area and depth of the plate interface at this location (Kita, Okada, Hasegawa, Nakajima, & Matsuzawa, 2010). Focal depths of 24 km and 7 km were taken for the 2011 CE ($M_w$ 9.1) earthquake (Yoshida, Ueno, Muto, & Aoki, 2011) and 1896 CE (Mw 8) earthquake (Satake, Fujii, & Yamaki, 2017). For the 1994 CE ($M_w$ 7.7) earthquake, a focal depth of 10 km was taken (Nagai, Kikuchi, & Yamanaka, 2001) which was also used as an estimation for the 1931 CE ($M_w$ 7.8) and 1968 CE ($M_w$ 8.2) earthquakes as all ruptured the same asperity. As magnitude saturation of strong ground motion takes place for earthquakes larger than $M_w$ 8.3, the magnitude of the 2011 CE earthquake is substituted with $M_w$ 8.4 (Usami et al., 2018).

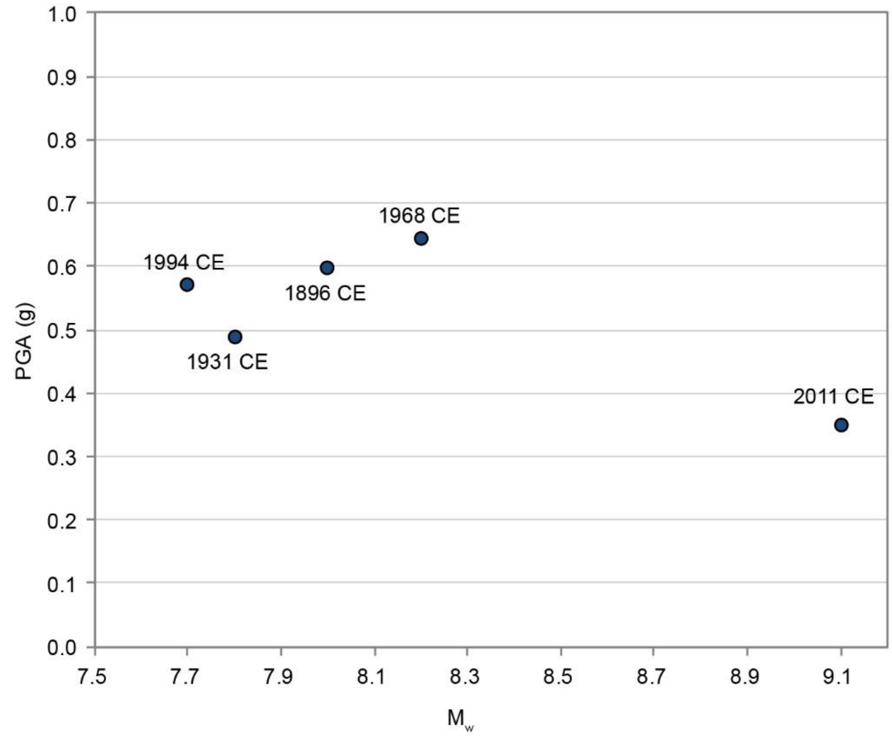


**Figure S1.** PGA versus $M_w$ for the five largest regional earthquakes.

| # | Earthquake | Date | $M_w$ | Focal depth (km) | Fault distance (km) | PGA (g) |
|---|---|---|---|---|---|---|
| 1 | 2011 CE Tohoku-oki | 11.03.2011 | 9.1 | 24 | 67 | 0.35 |
| 2 | 1994 CE Sanriku-oki | 28.12.1994 | 7.7 | 10 | 14 | 0.57 |
| 3 | 1968 CE Tokachi-oki | 16.05.1968 | 8.2 | 10 | 14 | 0.64 |
| 4 | 1931 CE Sanriku-oki | 09.03.1931 | 7.8 | 10 | 22 | 0.49 |
| 5 | 1896 CE Sanriku-oki | 15.06.1896 | 8 | 7 | 14 | 0.60 |

**Table S1.** Date, $M_w$, focal depth and fault distance of the five largest earthquakes along with calculated PGA.

**Dataset S1.** Measurement values of core GeoB21818-2 for radiodensity, cumulative grainsize, undrained shear strength, xs$^{210}$Pb activity, $^{137}$Cs activity and age as calculated by the xs$^{210}$Pb age-depth model.

**References**


Arnaud, F., Lignier, V., Revel, M., Desmet, M., Beck, C., Pourchet, M., . . . Tribovillard, N. (2002). Flood and earthquake disturbance of 210Pb geochronology (Lake Anterne, NW Alps). *Terra Nova*, *14*(4), 225–232. https://doi.org/10.1046/j.1365-3121.2002.00413.x

Cundy, A. B., & Croudace, I. W. (1995). Physical and chemical associations of radionuclides and trace metals in estuarine sediments: an example from Poole Harbour, Southern England. *Journal of Environmental Radioactivity*, *29*(3), 191–211. https://doi.org/10.1016/0265-931X(95)00031-5

Kita, S., Okada, T., Hasegawa, A., Nakajima, J., & Matsuzawa, T. (2010). Anomalous deepening of a seismic belt in the upper-plane of the double seismic zone in the Pacific slab beneath the Hokkaido corner: Possible evidence for thermal shielding caused by subducted forearc crust materials. *Earth and Planetary Science Letters*, *290*(3-4), 415–426. https://doi.org/10.1016/j.epsl.2009.12.038

Nagai, R., Kikuchi, M., & Yamanaka, Y. (2001). Comparative Study on the Source Processes of Recurrent Large Earthquakes in Sanriku-oki Region: the 1968 Sanriku-oki Earthquake. *Zisin(2)*, *54*, 267–280. https://doi.org/10.4294/zisin1948.54.2_267

Sanchez-Cabeza, J. A., & Ruiz-Fernández, A. C. (2012). 210Pb sediment radiochronology: An integrated formulation and classification of dating models. *Geochimica Et Cosmochimica Acta*, *82*, 183–200. https://doi.org/10.1016/j.gca.2010.12.024

Satake, K., Fujii, Y., & Yamaki, S. (2017). Different depths of near-trench slips of the 1896 Sanriku and 2011 Tohoku earthquakes. *Geoscience Letters*, *4*, 33. https://doi.org/10.1186/s40562-017-0099-y

Si, H., & Midorikawa, S. (1999). New attenuation relationships for peak ground acceleration and velocity considering effects of fault type and site condition. *Journal of Structural and Construction Engineering (Transactions of AIJ)*, *64*(523), 63–70. https://doi.org/10.3130/aijs.64.63_2

Usami, K., Ikehara, K., Kanamatsu, T., & McHugh, C. M. (2018). Supercycle in great earthquake recurrence along the Japan Trench over the last 4000 years. *Geoscience Letters*, *5*, 11. https://doi.org/10.1186/s40562-018-0110-2

Yoshida, Y., Ueno, H., Muto, D., & Aoki, S. (2011). Source process of the 2011 off the Pacific coast of Tohoku Earthquake with the combination of teleseismic and strong motion data. *Earth, Planets and Space*, *63*(7), 565–569. https://doi.org/10.5047/eps.2011.05.011
